# Management of Retrosternal Goiter in Resource-Limited Settings: Outcomes From 28 Cases Using Cervical Approach

**DOI:** 10.7759/cureus.41288

**Published:** 2023-07-02

**Authors:** Saif A Ghabisha, Faisal Ahmed, Saleh Al-wageeh, Qasem Alyhari, Mohamed A Badheeb, Abdulfattah Altam, Afaf Alsharif

**Affiliations:** 1 Department of General Surgery, School of Medicine, Ibb University of Medical Science, Ibb, YEM; 2 Department of Urology, Ibb University, Ibb, YEM; 3 Department of General Medicine, King Khalid Hospital, Najran, SAU; 4 Department of General Surgery, 21 September University, Sana'a, YEM; 5 Department of Gynecology, Jeblah University for Medical and Health Sciences, Ibb, YEM

**Keywords:** retrosternal, thyroidectomy, postoperative complication, goiter, cervical approach

## Abstract

Background

Despite thyroidectomy being the preferred approach for retrosternal goiter (RSG), controversies surround its rationale in asymptomatic cases. This study aimed to investigate the treatment of RSG in resource-limited settings.

Methods

A retrospective study conducted between April 2010 and June 2022 included 28 RSG cases who underwent thyroidectomy using the cervical approach at Al-Nasar Hospital, Ibb, Yemen. A bivariate analysis was performed to investigate the risk factors for postoperative complications.

Results

The main age was 49.4±9.9 years, and most of them (60.7%) were females. The main symptoms were cervical mass appearance and breathing difficulty in 75 %, and 32.1%, respectively. Twenty-four (86%) cases were classified as Grade 1 (above aortic arch) and four (14%) cases were classified as Grade 2 (aortic arch to the pericardium). All patients underwent total thyroidectomy through the cervical approach without needing sternotomy. The mean operative time was 121.9±26.7min (99-200 min) and the mean intraoperative bleeding was 321.2±137.4 mL. Postoperatively, the malignant entity was histopathologically proven in seven patients (25%). The postoperative complications (14%) were transient hypocalcemia in two (7.1%) and hematoma in two (7.1%). Older age, bigger thyroid mass, extension below the aortic arch (Grade 2), longer operative time and bleeding, intensive care unit admission, and malignant features are associated with postoperative complications (all p < 0.05).

Conclusion

Cervical approach for patients with RSG in our experience is an optimum, feasible, and less invasive surgical approach, in a resource-limited setting. Older age, bigger thyroid, extension below the aortic arch, longer operative time and bleeding, intensive care unit admission, and malignant features are associated with postoperative complications.

## Introduction

Goiter, a Latin origin term, refers to a doubling in thyroid size or weight over 40g [1]. Retrosternal goiter (RSG) is characterized by its variable definition, often referring to a goiter that extends below the thoracic inlet or has more than 50% of its volume below this level [2]. The reported incidence of RSG varies drastically due to the lack of defining criteria, with a reported incidence of 2%-26% of thyroidectomy patients [3,4]. While RSG may remain asymptomatic for an extended period, the risk of sudden growth and extrinsic airway compromise due to bleeding, cystic degeneration, or malignant transformation can be life-threatening [3,4]. Consequently, surgeons treating thyroid disorders face unique challenges associated with RSG, both in determining the appropriate indications for surgery preoperatively and in achieving the safe removal of intrathoracic goiters with minimal morbidity during the operation [5].

There is substantial disagreement regarding the selection of RSG patients for surgery. While the majority of endocrine surgeons consider retrosternal extension as an independent absolute indication for thyroidectomy, others argue that only patients with symptoms or suspected malignancy should undergo surgery [3,4]. In Battistella et al.'s study, about 2% of patients with RSG may present with acute respiratory failure and will need emergency surgery, as well as 1.5% may present with vein obstruction [6]. Furthermore, the increased use of radiological investigations has revealed a large number of RSG patients who are frequently asymptomatic [5]. Many surgical approaches and strategies exist, including but not limited to cervical neck incision, combined thoracic incision, or thoracic midline incision [4,6]. Fortunately, in the majority of cases of RSG, a cervical incision alone is usually adequate. However, in specific cases, an accessory incision, sternotomy, or other surgical procedures may be required [7].

In resource-limited settings, there is insufficient information available regarding the extent and characteristics of RSG [8]. A previous report from our hospital showed promising results with a cervical approach for the treatment of huge RSG in a 64-year-old man [9]. This study aimed to evaluate the feasibility of total thyroidectomy via a cervical incision for RSG cases at a single institute in Yemen, considering the limitations in resources and general surgeons as the primary operators. Moreover, we aimed to contribute a broader perspective on thyroidectomy in a data-limited nation where thyroid disorders are diagnosed clinically and managed exclusively by general surgeons.

## Materials and methods

Study design and setting

A case series with a retrospective chart review was conducted to include 28 cases diagnosed with RSG and underwent thyroidectomy using the cervical approach at AL Nasar Hospital, Ibb, Yemen between April 2010 and June 2022. The Ethics Research Committees of Al-Nasar Hospital provided their approval for the study (ID: 2723), which adhered to the ethical principles outlined in the Declaration of Helsinki.

Data collection

**Figure 1:**
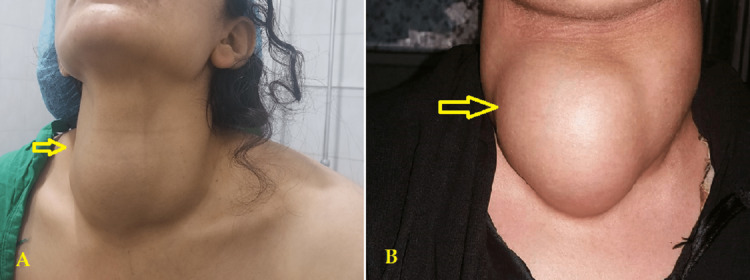
Preoperative photo showing the thyroid mass (arrow) in (A) female patient and (B) male patient.

Demographic data, including age and gender, as well as clinical presentation factors such as the presence of a neck mass (Figures 1A, 1B), breathing difficulty, pain, and dysphagia, were examined in the analysis of patient records.

**Figure 2:**
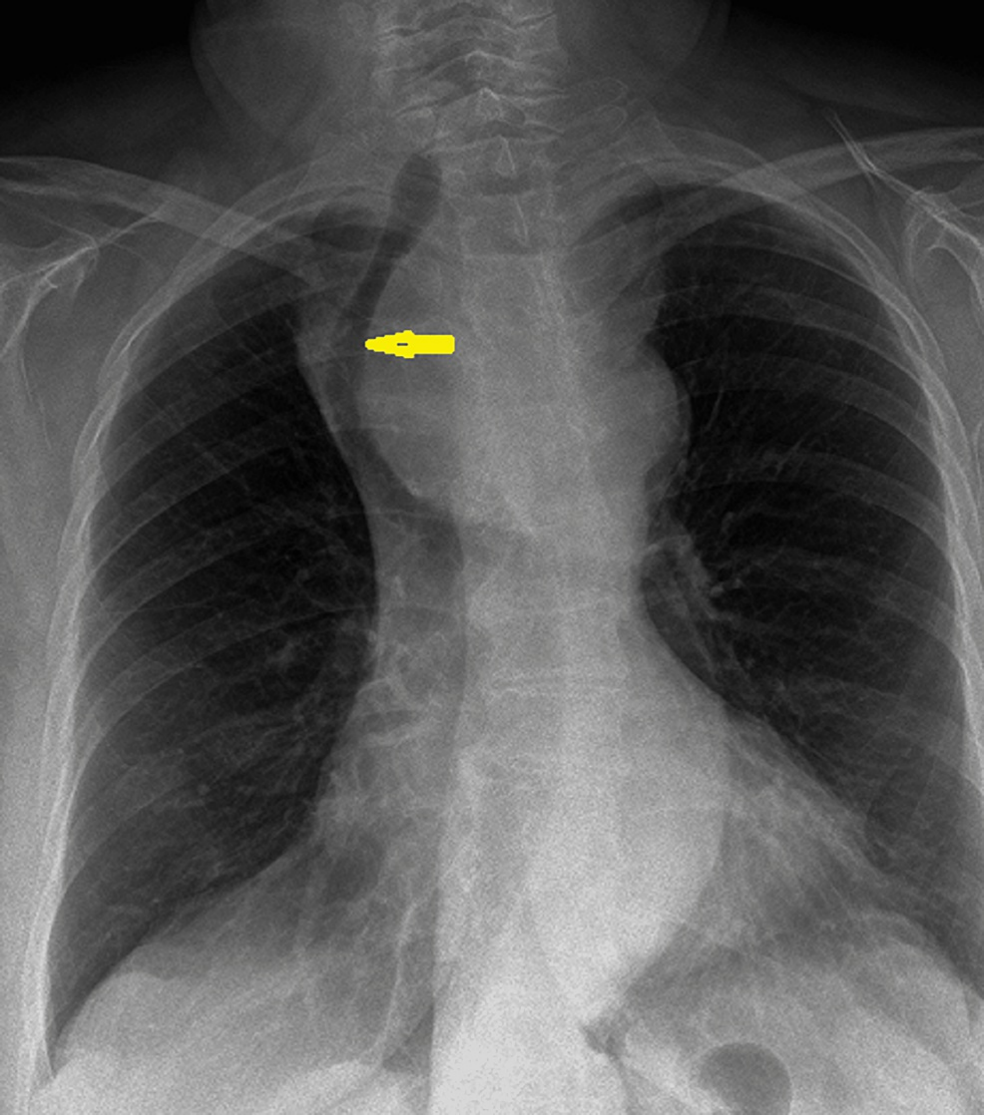
Radiography x-ray showing retrosternal thyroid mass causing tracheal deviation (arrow).

Radiological imaging findings from plain radiology, ultrasonography, and computed tomography (CT) scans were also reviewed (Figure 2).

**Figure 3:**
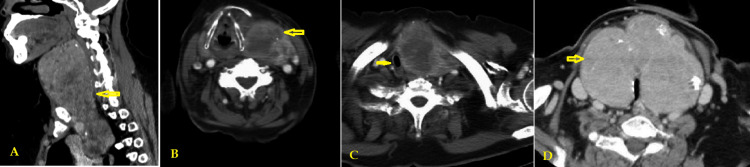
Computed tomography scan showing retrosternal thyroid mass (A) lateral view (arrow), (B) transverse section (arrow), (C) mass causing tracheal deviation (arrow), (D) big thyroid mass with calcifications (arrow).

Additional aspects evaluated included the classification of RSG, the requirement for supplementary procedures such as sternotomy or manubriotomy, operative time, blood loss, the need for intensive care unit (ICU) admission, postoperative complications, and the duration of clinical follow-up (Figures 3A-3D).

Main outcome

The primary outcome is to report our findings. The secondary outcome is to find the risk factors associated with postoperative complications.

Definition

The grading and anatomical classification system developed by Huins were employed to categorize cases of RSG. This classification system involved three grades: Grade 1 referred to goiters located above the level of the aortic arch (above T4), Grade 2 represented goiters extending from the aortic arch to the pericardium, and Grade 3 encompassed goiters located below the right atrium. The diagnosis of RSG was made when the preoperative CT scan report indicated any extension of the goiter into the thorax through the thoracic inlet [10,11].

Statistical analysis

The statistical analysis was performed using IBM SPSS software (version 22, IBM Corp., Armonk, NY). Descriptive statistics were calculated, presenting variables as means with corresponding standard deviations. Frequencies were reported using descriptive analysis to summarize categorical variables. The normality of the data was assessed using the Smirnov-Kolmogorov test. To identify factors associated with postoperative complications, quantitative variables were compared using either the independent samples T-test or the Mann-Whitney U test, depending on the normality of the data. The Chi-square test or Fisher's exact test was utilized for qualitative variables to examine associations with postoperative complications. These statistical tests were employed to determine the significance of relationships between variables and postoperative complications in the study population.

## Results

**Figure 4:**
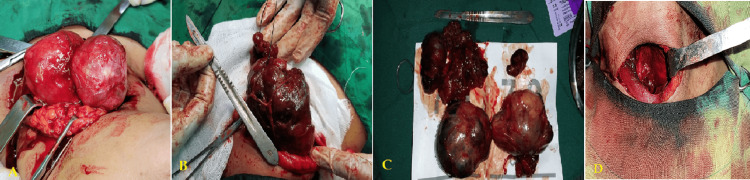
Intraoperative photos showing (A) cervical approach, (B) big thyroid mass, (C) mass after removal, and (D) site of operation postoperatively.

The main age was 49.4±9.9 years (range 34-69 years), and most of them (60.7%) were females. The mean duration of symptoms presentation was 25.1±13.7 months (range 5-54 months) and the main symptoms were cervical mass appearance and breathing difficulty in 75%, and 32.1%, respectively. Regarding the classification type of RSG, 24 (86%) cases had a Grade 1 (above aortic arch (above T4)) and 4 (14%) cases had Grade 2 (aortic arch to the pericardium), while no patient had an RSG Grade 3 (below right atrium). All the patients underwent a total thyroidectomy without needing sternotomy or manubriotomy (Figures 4A-4D).

The mean operative time was 121.9±26.7 min (range 99-200) and the mean intraoperative bleed loss was 321.2±137.4 (range 110 - 650) ml. The mean of hospital stay was 5.3 ± 1.1day (Median: 5 (Min: 4 to Max: 8)). Postoperatively, the malignant entity was histopathologically proven in seven patients (25%). The total postoperative complications rate was 14.2% (transient hypocalcemia in 7.1% and hematoma in 7.1%). Within a follow-up of 13.4±6.5 months (Median: 12.5 (Min: 2 to Max: 30)), no symptoms of recurrence were observed. The patient characteristics, operative findings, and complications are summarized in Table 1.

**Table 1:** Patient characteristics

Variables	N (%)
Age (year), (Mean ±SD)	49.4 ±9.9 (range 34 - 69)
Gender	
Male	11 (39.3)
Female	17 (60.7)
Symptoms	
Cervical swelling	21 (75.0)
Breathing difficulty	9 (32.1)
Neck discomfort	7 (25.0)
Palpable mass during an examination	7 (25)
Symptoms duration (months), Mean ±SD	25.1±13.7 (range 5-54)
Retrosternal grade	
Grade 1 (Above aortic arch)	24 (85.7)
Grade 2 (Extension beyond the Carina)	4 (14.3)
Thyroid size (ml), Mean±SD	133.0 ±37.8 (range 70-215)
Operative time (min), Mean±SD	121.9±26.7 (range 99-200)
Tracheal compression	22 (78.6)
Operative blood loss (mL), Mean±SD	321.2±137.4 (range 110-650)
Hospital stays (day), Mean±SD	5.3 ±1.1 (range 4-8)
Need intensive care unit	8 (28.6)
Complications	4 (14.2)
Type of complication	
Transnet hypocalcemia	2 (7.1)
Post-operative hematoma	2 (7.1)
Histopathologic findings	
Multi-nodular goiter	21 (75.0)
Papillary thyroid carcinoma	6 (21.4)
Follicular thyroid carcinoma	1 (3.6)
Follow-up (months), Mean±SD	13.4 ±6.5 (range 2-30)

Factors associated with postoperative complications

Older age, bigger thyroid mass, extension below the aortic arch (Grade 2), longer operative time, significant intraoperative bleeding, ICU admission, and malignant features were associated with postoperative complications (all p < 0.05) (Table 2).

**Table 2:** Bivariate analysis of risk factors for postoperative complications of total thyroidectomy for substernal goiter

Variables	Subgroups	Complications, N (%)		P-value
		No (N=24)	Yes (N=4)	
Age (year)	Mean (SD)	46.7 (7.7)	65.8 (2.2)	<0.001
Gender	Male	9 (37.5)	2 (50.0)	1.000
	Female	15 (62.5)	2 (50.0)	
Symptoms duration (months)	Mean (SD)	24.4 (14.6)	29.2 (4.9)	0.520
Thyroid size (ml)	Mean (SD)	120.4 (22.5)	208.8 (6.3)	<0.001
Operative time (min)	Mean (SD)	111.8 (7.8)	182.5 (15.0)	<0.001
Operative blood loss (ml)	Mean (SD)	278.9 (90.2)	575.0 (86.6)	<0.001
Hospital stays (day)	Mean (SD)	5.3 (1.2)	5.5 (0.6)	0.738
Need intensive care unit	No	20 (83.3)	0 (0.0)	0.005
	Yes	4 (16.7)	4 (100.0)	
Retrosternal grade	Grade 1	24 (100.0)	0 (0.0)	<0.001
	Grade 2	0 (0.0)	4 (100.0)	
Histopathologic findings	Benign	21 (87.5)	0 (0.0)	0.002
	Malignant	3 (12.5)	4 (100.0)	

## Discussion

In this study, we report our experiences in the surgical management of RSG in a resource-limited setting. Additionally, we studied the factors of postoperative complications. These included advanced age, larger thyroid size, extension beyond the carina, longer operative time and bleeding, ICU admission, and malignant histopathological features. The development of goiter is believed to have a multifactorial etiology. However, the enlargement of the thyroid gland per se can be seen as an adaptive response to decreased thyroid hormone production. Consequently, various etiologic entities can contribute to the development of goiter [2]. In developed countries, inflammatory conditions, such as Hashimoto thyroiditis and postpartum thyroiditis, as well as infiltrative disorders like sarcoidosis, may be considered important causes of goiter. Iodine deficiency remains the most prevalent cause, of goiter in limited-income countries and worldwide [12].

There is considerable controversy regarding the RSG definition, while various definitions had been proposed, the practicality and the suitability of each of them remain highly subjective. Hinus et al. introduced a definition that includes goiter extension below the thoracic inlet (greater than 3 cm), the proportion of the goiter located within the chest (at least 50%), and the presence of glandular spread into the mediastinum below the thoracic inlet [11]. In this study, RSG was outlined based on the expansion of the mass below the thoracic inlet level, consistent with the Huins grading system [11]. This approach is supported by previous studies conducted by Nakaya et al. and Anikin et al. [4,13].

Indeed, the proximity of RSG to vital mediastinal structures renders it a challenging, or even life-threatening illness [10]. Consensus exists that medical treatments are generally ineffective in treating RSG [1,14]. Thyroidectomy is widely recognized as an important part of the thyroid disease management pathway. Nevertheless, a lack of agreement exists regarding the specific indications that warrant the implementation of thyroidectomy [10]. Although the mere presence of RSG warrants surgery by many surgeons. some symptomatic cases may present with acute complications that necessitate immediate surgery, which could be challenging to untrained staff. The other issue is the risk of harboring malignancy by RSG, as the new intra-thoracic environment may trigger malignant transformation, in this series, the authors present a rate of 25% of malignancy which is quite high [1,6,15]. A few others believe that surgery should be reserved for symptomatic patients or when there is a high susception of malignancy [3,4]. In this study, the main symptoms observed were the emergence of a cervical mass and breathing difficulties, reported in 75% and 32.1% of cases, respectively, aligning with previous findings [1,6,14,15]. The enlargement of the goiter can lead to compression of the esophagus, vascular and neurologic structures (e.g., recurrent laryngeal nerves), or in airways in more severe cases, necessitating urgent surgical intervention [6].

Commonly used preoperative imaging modalities for assessing retrosternal goiter (RSG) include chest x-ray, thyroid ultrasound, CT, and scintigraphy [10]. Ultrasonography is easily accessible, low-risk, and allows for cytological evaluation through fine-needle aspiration cytology (FNAC). However, it has limitations in accurately assessing retrosternal extension. Additionally, there are various technical difficulties that limit the accuracy of FNAC in the mediastinal region [10]. Thus, we did not perform FNAC in all cases of our study. CT scan is considered the most suitable method for determining the extent of the goiter, providing detailed anatomical information of the thyroid gland, trachea, and esophagus. Nevertheless, the routine use of iodinated radiocontrast agents should be avoided as they may induce overt hyperthyroidism [10,16].

Surgical excision of RSG is predominantly accomplished via a cervical approach in the majority of patients [10]. In our study, all the patients underwent total thyroidectomy through a cervical approach. This approach was endorsed vastly for various reasons. First, the highly vascular mediastinal structures increase the risk of bleeding in an anatomically constrained area [7]. Secondly, most of the RSG resides on the anterior mediastinum, making extra-cervical approaches of a limited need, primarily with posterior RSG that presents in 10% of cases [10,17]. Thirdly, unless there is consideration of malignancy, the cervical approach allows for total or partial thyroidectomy [10,18]. Nonetheless, there are few reports in the literature, in which non-cervical approaches were implemented in a set of patients. This included thoracotomy or sternotomy, each of these were usually indicated for external factors including inaccessible masses, invasive malignancy, adherence to adjacent structures, re-operation, or emergency surgery, these cases were reviewed and summarized by White et al. [1].

The post-operative complications of RSG are not uncommon, transient hypocalcemia was observed in up to 15% of cases [19]. Vocal alterations and nerve palsies are noticed more frequently in posterior RSG, these cases are often associated with invasive malignancies, which generally have a poorer prognosis [10]. In our study, we encountered an overall complication rate of 14.2%, this relatively low previous reports may have been influenced by the fact that most cases were located in the anterior mediastinum. This location is less likely to disturb the anatomical relationship between the parathyroid glands and the recurrent laryngeal nerve, which both reside in the tracheoesophageal groove. Notably, there were no cases of wound infection or other injuries reported, suggesting that RSG does not pose a greater risk compared to cervical goiter excision. This finding aligns with reports by Raffaelli et al. and Abdelrahman et al. [10,20].

Compared to cervical goiters, there is currently no compelling evidence to support a higher occurrence of incidental thyroid cancer in RSG cases. However, the potential increased risk of malignancy, in addition to the limitations in ruling out malignant nature of the RSG are often cited as a justification for considering prophylactic thyroidectomy in RSG management [21]. The reported incidence of malignancy in cervical and intrathoracic goiters varies across the literature, ranging from 3% to as high as 35% [22,23]. In our study, 25% of patients (seven individuals) were diagnosed with thyroid malignancy based on the final pathological analysis, with papillary carcinoma being the predominant histologic subtype. This finding aligns with previously published reports [21,24].

The factors pertaining to postoperative complications in RSG exhibit variability and may be reported differently across various studies. These discrepancies can primarily be attributed to the limitations imposed by small sample sizes and the heterogeneity of populations included in these studies. Studies reported discrepancies related to the surgery and the surgical team's experience, or intrinsic characteristics of the goiter [18,20]. A retrospective series presented by Cichon et al., involving 88 patients with RSG, analyzed the high-risk group predisposed to sternotomy [25]. The study's findings indicated that recurrent goiter, posterior mediastinal location, aberrant adenoma, and mediastinal blood supply were statistically significant risk factors for sternotomy. However, it is important to acknowledge the limitations of this series, as it relied on a retrospective design, lacked randomization, and did not include a control group [25].

Our study identified several factors associated with postoperative complications in RSG. These included advanced age, larger thyroid size, extension beyond the carina, longer operative time and bleeding, ICU admission, and malignant histopathological features.

It has been observed that advanced age is associated with longer surgical durations and the need for larger tissue resections. While these factors are not exclusively limited to older patients, there is a clear correlation between age-related factors and an increased risk of complications [26,27]. It is crucial, however, to acknowledge that thyroidectomies for RSG carries significant morbidity and mortality rates, especially among elderly patients. Therefore, careful consideration of the risks and benefits is essential before deciding to proceed with surgery [5]. In our study, we found that larger RSG and infra-cranial extension of RSG were linked to higher post-operative complications. These findings are consistent with previous reports that have observed higher mortality and morbidity rates associated with larger goiter sizes. Although it remains unclear whether size can be considered an independent predictor of postoperative complications, it is evident that larger goiters can hinder intubation, cause upper airway obstruction, complicate the operative approach, and prolonged operative time [28]. Specifically, RSG thyroidectomies were frequently associated with longer operative times, which were found to be correlated with worse postoperative outcomes in our study. However, there is currently insufficient substantial evidence to support a direct association between operative time and higher rates of postoperative complications [29]. 

Notably, our study identified a significant association between intraoperative bleeding, admission to the ICU, and malignancy with higher rates of postoperative complications. While these findings have been reported in previous research [3,30]. To the best of our knowledge, no study has effectively controlled for these variables in relation to size, extension, or the specific presentation of these cases. As RSG can often manifest in advanced stages with posterior extensions, which adds complexity to the surgical management. Further studies are required to comprehensively elucidate the potential relationship between these variables and their impact on post-operative complications.

Several limitations must be considered when interpreting the findings of the present study. Firstly, the retrospective design introduces limitations on documentation and data collection. Additionally, the relatively smaller sample size might restrict the statistical power, limiting the ability to draw robust conclusions or generalize the findings to larger populations. Furthermore, a significant limitation is the absence of a clear definition or standardized criteria for RSG which may introduce variations in patient selection and outcome assessment. Moreover, the lack of data on familial history or prior diseases in the analyzed cohort poses limitations on understanding potential risk factors and their impact on outcomes. Another notable limitation is the absence of documented preoperative diagnoses of malignant RSG using FNAC. The challenges associated with obtaining representative samples due to the size and localization of the gland within the thoracic cavity likely contributed to the lack of available data. Furthermore, the limited accuracy of the FNAC procedure itself may have hindered the preoperative diagnosis of malignant RSG.

## Conclusions

Based on our experience, the cervical approach is considered an optimal, feasible, and minimally invasive surgical approach for patients with RSG, even in resource-limited settings. Postoperative complications are associated with several factors, including older age, larger thyroid size, extension below the aortic arch, longer operative time, increased intraoperative bleeding, ICU admission, and the presence of malignant features.

## References

[1] White ML, Doherty GM, Gauger PG (2008). Evidence-based surgical management of substernal goiter. World J Surg.

[2] Khairy GA, Al-Saif AA, Alnassar SA, Hajjar WM (2012). Surgical management of retrosternal goiter: local experience at a university hospital. Ann Thorac Med.

[3] Di Crescenzo V, Vitale M, Valvano L (2016). Surgical management of cervico-mediastinal goiters: our experience and review of the literature. Int J Surg.

[4] Nakaya M, Ito A, Mori A (2017). Surgical treatment of substernal goiter: an analysis of 44 cases. Auris Nasus Larynx.

[5] Landerholm K, Järhult J (2015). Should asymptomatic retrosternal goitre be left untreated? A prospective single-centre study. Scand J Surg.

[6] Battistella E, Pomba L, Sidoti G, Vignotto C, Toniato A (2022). Retrosternal goitre: anatomical aspects and technical notes. Medicina (Kaunas).

[7] Wang X, Zhou Y, Li C (2020). Surgery for retrosternal goiter: cervical approach. Gland Surg.

[8] Ghabisha S, Ahmed F, Alyhari Q (2022). Assessment of demographic characteristics and histopathological pattern of thyroidectomies patients in a resource-limited setting: a retrospective cross-sectional study. Pan Afr Med J.

[9] Ghabisha S, Ahmed F, Eslahi A (2021). A case report of a huge euthyroid goiter with retrosternal extension. JEMATC.

[10] Abdelrahman H, Al-Thani H, Al-Sulaiti M, Tabeb A, El-Menyar A (2020). Clinical presentation and surgical treatment of retrosternal goiter: a case series study. Qatar Med J.

[11] Huins CT, Georgalas C, Mehrzad H, Tolley NS (2008). A new classification system for retrosternal goitre based on a systematic review of its complications and management. Int J Surg.

[12] Knobel M (2016). Etiopathology, clinical features, and treatment of diffuse and multinodular nontoxic goiters. J Endocrinol Invest.

[13] Anikin V, Welman K, Asadi N, Dalal P, Reshetov I, Beddow E (2021). Retrosternal goiter in thoracic surgical practice. Khirurgiia (Mosk).

[14] Flati G, De Giacomo T, Porowska B (2005). Surgical management of substernal goitres. When is sternotomy inevitable?. Clin Ter.

[15] Unlu MT, Aygun N, Kostek M, Isgor A, Uludag M (2022). Substernal goiter: from definitions to treatment. Sisli Etfal Hastan Tip Bul.

[16] Rieu M, Bekka S, Sambor B, Berrod JL, Fombeur JP (1993). Prevalence of subclinical hyperthyroidism and relationship between thyroid hormonal status and thyroid ultrasonographic parameters in patients with non-toxic nodular goitre. Clin Endocrinol (Oxf).

[17] Hashmi SM, Premachandra DJ, Bennett AM, Parry W (2006). Management of retrosternal goitres: results of early surgical intervention to prevent airway morbidity, and a review of the English literature. J Laryngol Otol.

[18] Li W, Li H, Zhang S, Tao Y, Wang X, Cheng J (2020). To explore the risk factors and preventive measures affecting the treatment of retrosternal goiter: an observational study. Medicine (Baltimore).

[19] Abboud B, Sleilaty G, Mallak N, Zeid HA, Tabchy B (2010). Morbidity and mortality of thyroidectomy for substernal goiter. Head Neck.

[20] Raffaelli M, De Crea C, Ronti S, Bellantone R, Lombardi CP (2011). Substernal goiters: incidence, surgical approach, and complications in a tertiary care referral center. Head Neck.

[21] Hardy RG, Bliss RD, Lennard TW, Balasubramanian SP, Harrison BJ (2009). Management of retrosternal goitres. Ann R Coll Surg Engl.

[22] Agarwal G, Aggarwal V (2008). Is total thyroidectomy the surgical procedure of choice for benign multinodular goiter? An evidence-based review. World J Surg.

[23] Nixon IJ, Simo R (2013). The neoplastic goitre. Curr Opin Otolaryngol Head Neck Surg.

[24] Erbil Y, Bozbora A, Barbaros U, Ozarmağan S, Azezli A, Molvalilar S (2004). Surgical management of substernal goiters: clinical experience of 170 cases. Surg Today.

[25] Cichoń S, Anielski R, Konturek A, Baczyński M, Cichoń W, Orlicki P (2008). Surgical management of mediastinal goiter: risk factors for sternotomy. Langenbecks Arch Surg.

[26] Ríos A, Rodríguez JM, Galindo PJ, Canteras M, Parrilla P (2005). Surgical treatment for multinodular goitres in geriatric patients. Langenbecks Arch Surg.

[27] Mekel M, Stephen AE, Gaz RD, Perry ZH, Hodin RA, Parangi S (2009). Thyroid surgery in octogenarians is associated with higher complication rates. Surgery.

[28] Blank RS, de Souza DG (2011). Anesthetic management of patients with an anterior mediastinal mass: continuing professional development. Can J Anaesth.

[29] Bove A, Di Renzo RM, D'Urbano G, Bellobono M, D'Addetta V, Lapergola A, Bongarzoni G (2016). Preoperative risk factors in total thyroidectomy of substernal goiter. Ther Clin Risk Manag.

[30] Chen Q, Su A, Zou X (2022). Clinicopathologic characteristics and outcomes of massive multinodular goiter: a retrospective cohort study. Front Endocrinol (Lausanne).

